# Immediate and Early Postnatal Care for Mothers and Newborns in Rural Bangladesh

**Published:** 2006-12

**Authors:** Uzma Syed, Sk. Asiruddin, Md. S.I. Helal, Imteaz I. Mannan, John Murray

**Affiliations:** ^1^ Saving Newborn Lives, Save the Children-USA, 2000 M St. NW, Suite 500, Washington, DC 20036, USA; ^2^ Saving Newborn Lives, Save the Children USA, Bangladesh Country Office, House 1A, Road 91, Gulshan 2, Dhaka 1212, Bangladesh

**Keywords:** Obstetric care, Postnatal care, Danger signs, Pregnancy, Community health workers, Impact studies, Bangladesh

## Abstract

The study evaluated the impact of essential newborn-care interventions at the household level in the Saving Newborn Lives project areas. Two household surveys were conducted following the 30-cluster sampling method using a structured questionnaire in 2002 (baseline) and 2004 (endline) respectively. In total, 3,325 mothers with children aged less than one year in baseline and 3,110 mothers in endline from 10 sub-districts were interviewed during each survey. The proportion of newborns dried and wrapped immediately after birth increased from 14% in 2002 to 55% in 2004; 76.2% of the newborns were put to the mother's breast within one hour of birth compared to 38.6% in baseline. Newborn check-up within 24 hours of delivery increased from 14.4% in 2002 to 27.3% in 2004. Postnatal check-up of mothers by trained providers within three days of delivery rose from 2.4% in 2002 to 27.3% in 2004. Knowledge of the mothers on at least two postnatal danger signs increased by 17.2%, i.e. from 47.1% in 2002 to 64.3% in 2004. Knowledge of mothers on at least three postnatal danger signs also showed an increase of 16%. Essential newborn-care practices, such as drying and wrapping the baby immediately after birth, initiation of breastmilk within one hour of birth, and early postnatal newborn check-up, improved in the intervention areas. Increased community awareness helped improve maternal and newborn-care practices at the household level. Lessons learnt from implementation revealed that door-to-door visits by community health workers, using community registers as job-aids, were effective in identifying pregnant women and following them through pregnancy to the postnatal periods.

## INTRODUCTION

Despite improvement in the infant and child health status, a high number of neonatal deaths (41 per 1,000 livebirths) pose a serious public-health concern in developing countries, including Bangladesh. Globally, almost three-quarters of neonatal deaths occur within the first seven days of delivery (1). However, there is a significant break in the continuum of care in the service-delivery strategy. The burden of maternal complications and deaths is also highest in the first few days of delivery (2, 3). Thus, immediate and early postnatal interventions (defined to be from delivery to first seven days), have the potential to change the maternal and child mortality scenario significantly in Bangladesh.

In Bangladesh, the perinatal (stillbirths and early neonatal) and late neonatal mortality scenario reflects a dreadful picture. To address the problem, governmental and non-governmental organizations (NGOs) have been implementing various interventions. The Saving Newborn Lives (SNL), an international initiative funded by the Bill and Melinda Gates Foundation, was launched in 2000 to improve the health of newborns globally. Its overall goal is to improve neonatal health and survival. The primary strategic objective to achieve this goal was to increase and sustain key health-practices and the use of essential services in communities. The activities of the programme focused on five intermediate results: strengthen and expand proven newborn health interventions; adapt and refine promising model programmes; advance the state of the art relating to newborn health; mobilize commitment and resources; and establish strategic partnerships. The strategies of the programme included community-based interventions, including behaviour change communication; improving services through training on essential newborn care; monitoring and evaluation of programme activities; advocacy and research.

The community-based interventions were undertaken in 10 upazilas (sub-districts) of Bangladesh in collaboration with three NGOs—CARE Bangladesh, BRAC, and Bangladesh Population Health Consortium (BPHC). Essential newborn care was integrated into the ongoing interventions of the NGOs. The major components of the interventions included: increasing the coverage of health workers and community-based caregivers trained and competent in providing essential newborn care and promoting positive maternal and newborn-care practices. The projects targeted primarily pregnant mothers and family decision-makers, such as husbands, mothers-in-law, caregivers (both formal and non-formal), and village leaders. The primary activities for the programme included: training, service-delivery, behaviour change communication, and advocacy to improve care during delivery, postnatal and neonatal periods, and referral of sick newborns. The frontline health workers, paramedics, and local traditional birth attendants were trained on newborn care following the cadre-specific training modules. A behaviour change communications strategy was developed based on findings of formative research and interventions-targeted messages on key healthful behaviours, such as birth-preparedness, clean delivery, early and exclusive breastfeeding, immediate drying and warming, and major danger signs. The postnatal visit strategy included two or more contacts with the mother and newborn by the health workers at home within the first week of delivery with the first visit within three days. Programme planning, development of materials, implementation, and routine monitoring were carried out jointly by Save the Children-USA, the partner NGOs, and professional bodies to ensure adequate support and sustainability.

For monitoring the progress, a list of core indicators was developed as per the country context relating to maternal and newborn health (Box). A baseline survey was conducted in the project areas in 2002. This paper presents endline-survey analysis of the efforts of Save the Children-USA to promote healthful maternal and newborn-care practices in the SNL project areas (map).

Box. **SNL core indicators**

**Table:** 

Prenatal care
Who received at least 2 TT doses during the last pregnancy, by card only
Who received at least 3 TT doses during life (card and history)
Who received prenatal care 2 or more times by a trained provider during the last pregnancy
Who received prenatal care 4 or more times from a trained provider during the last pregnancy
Delivery care
With infant aged less than one year whose birth was attended by a skilled provider
With infant aged less than one year whose birth was attended by a trained provider
Who know at least two danger signs during labour and delivery
Maternal postnatal care
Who received postnatal care from trained provider within 3 days of delivery
Who received postnatal care from trained provider within 1 week of delivery
Who know at least 2 postnatal maternal danger signs after delivery
Newborn care
Who gave colostrum
Who breastfeed their infants within one hour of birth
Who did not give foods other than breastmilk in the first 3 days after birth
Children aged less than 1 month who breastfeed their children exclusively
Infant received newborn care from any provider within 3 days after delivery
Infant received newborn care from any provider within 1 week after delivery
Who know at least two newborn danger signs

SNL=Saving Newborn Lives;

TT=Tetanus toxoid

**Map.**
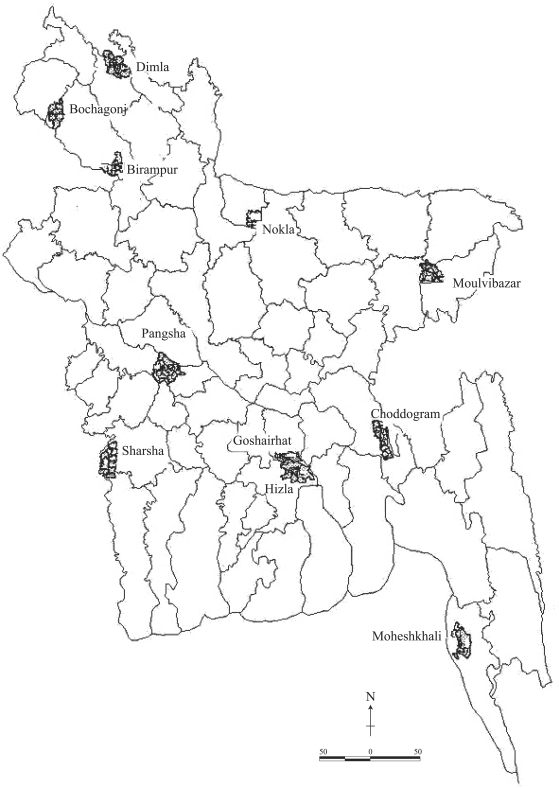
Saving Newborn Lives project areas

The main objectives of the endline survey were to (a) measure changes in the selected SNL indicators since the baseline survey of 2002 and (b) provide estimates on variables indicative of the status of maternal and neonatal care during prenatal, delivery and postnatal periods at the household level in the project areas.

Assessing programme performance requires looking at changes in health behaviours and health outcomes and distinguishing the contributions of the SNL project to these changes compared to contributions of others which may have led to these changes.

Analyzing the changes in health knowledge behaviours and practices from 2002 to 2004 and linking those changes to the presence and intensity of SNL efforts can evaluate the impact of the project in improving the maternal and newborn health status.

## MATERIALS AND METHODS

Associates for Community and Population Research (ACPR), Dhaka, conducted the two surveys. The surveys (baseline and endline) collected information using a structured multi-item questionnaire from mothers with children aged less than one year of the 10 SNL project upazilas served by its partner NGOs, such as BRAC, BPHC, and CARE Bangladesh, and Nokla upazila of Sherpur district (non-intervention area).

Both baseline and endline surveys used the 30-cluster sampling method. To minimize selection bias and for greater accuracy in measuring changes on important indicators, the same clusters used in the baseline survey were retained in the endline survey. Data were collected from Nokla to serve as a comparison area.

The surveys (baseline and endline) used a two-stage sampling method that was selected using available household data collected by the Bangladesh Bureau of Statistics (1991). A rural upazila is divided into unions and then into mauzas. An urban upazila is divided into wards and then mahallas. Thirty mauzas/mahallas were selected from each of the survey domains with probability proportionate to size (PPS) using the census frame of the respective upazila. A randomly-selected segment of approximately 120 households of a selected mauza/mahalla constituted a cluster. From each cluster, 12 mothers with children aged less than one year were selected using the systematic random procedure with the expectation that at least 10 respondents would be available for interview successfully from a cluster. Only one mother from a household was selected for interview. In total, 3,325 mothers in the baseline and 3,110 mothers in the endline survey from 10 upazilas were successfully interviewed.

A household schedule was used for collecting certain information, such as age and sex of household members [A household was defined as a person or group of people who live together and share same food]. This information was used for identifying eligible respondents in the household for random selection. The questionnaire that was used in the baseline survey was used in the endline survey with some minor modifications. It was pre-tested in a similar group of mothers outside the intervention area prior to its finalization. Ten teams—each comprising two female interviewers and one male supervisor—were deployed after an intensive training of nine days.

The processing operation consisted of office editing, coding of open-ended questions, data entry, and editing inconsistencies found by computer programmes. A data-entry package was developed in accordance with the data-collection instruments. Data were processed using the SPSS software (version 10.0). Results for all the project areas (combined) were estimated using appropriate weights.

## RESULTS

### General characteristics

Table 1 shows the distribution of mothers with children aged less than one year by selected background characteristics; 62.3% of the mothers of the SNL project areas were aged 20–34 years, 32.2% were aged less than 20 years, and 5.5% were aged 35 years or above. Their mean age was 23.2 years. The age distribution of the respondents of all the survey upazilas was similar. Most (99.9%) mothers were married. More than nine in every ten respondents were Muslims, with most of the remainder Hindus. The mean number of living children per respondent was 2.4. Table 1 also shows that 32.6% of the mothers in the project area had never attended any school, 10.6% had either attended or completed primary education, and 40.2% had some secondary or higher level of education.

**Table 1.** Percentage distribution of mothers with children aged less than one year, by background characteristics, according to the SNL project areas, 2004

Characteristics	SNL project areas	
	10 upazilas (intervention area)	1 upazila (non-intervention area)
Age (years)		
<20	32.2	33.1
20–34	62.3	61.3
35+	5.5	5.6
Mean age (years)	23.2	23.1
Highest level of education		
No education	32.6	52.6
Primary incomplete	16.6	14.2
Primary complete	10.6	12.7
Secondary +	40.2	20.4
Working status		
Working	9.9	9.3
Not working	90.1	90.7
Mean number of living children		
Son	1.1	1.20
Daughter	1.2	1.24
All	2.4	2.44
Number	2,787	323

SNL=Saving Newborn Lives

### Socioeconomic status

The mothers and their households were categorized into socioeconomic levels using an index of household assets. Asset information was used here in the absence of information on household expenditure and household income. The methodology was followed in the Bangladesh Demographic and Health Survey 1996–1997 and 1999–2000 (4, 5). Comparing asset quintiles, it was evident that, among the SNL project upazilas, populations of Moheshkhali, Dimla, and Bochagonj were relatively poorer than the populations of other upazilas (Table 2).

**Table 2.** Socioeconomic status in the SNL project areas, 2004

Characteristics	All projects	Moheshkhali	Dimla	Bochagonj
Asset quintile				
Poorest	20.4	45.2	34.5	34.3
2	18.4	15.0	19.7	17.8
3	19.6	16.5	21.3	19.5
4	20.6	15.9	14.5	11.6
Richest	21.0	7.5	10.0	16.8
Total	100.0	100.0	100.0	100.0
Number	2,787	321	310	303

SNL=Saving Newborn Lives

### Prevention of hypothermia

**Fig. 1.**
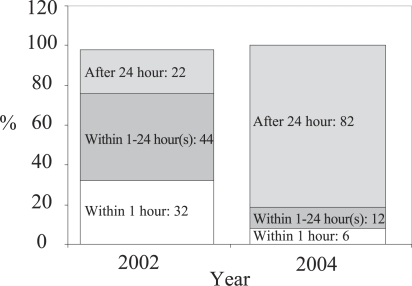
Bathing time of newborns after delivery in the SNL project areas by survey years

The proportion of newborns dried and wrapped immediately after birth increased from 14% in 2002 to 55% in 2004. This implies that interventions by trained traditional birth attendants had a positive impact. In 2002, 14% of 731 and 12% of 41 babies in the intervention and the non-intervention area respectively were dried and wrapped immediately after delivery. The corresponding figures in 2004 were 55% of 1,472 and 15% of 44 babies. Delaying bathing by 24 hours took place in 82% of births, showing a 62% increase from the baseline (22%) (Fig. 1).

### Breastfeeding

Mothers were asked questions relating to time of initiation, feeding of colostrums, and exclusive breastfeeding. Breastfeeding practices had improved substantially; practice of putting the newborn to the mother's breast immediately after birth increased from 38.6% in 2002 to 76.2% in 2004 (p=0.01) and was higher compared to the non-intervention area (Table 3). Most (96.3%) babies in the intervention area were put to the breast within the first day of life. Most (96.5%) mothers reported giving colostrum to the baby, and 81.2% did not feed anything before giving breastmilk.

**Table 3.** Percentage of mothers, by breastfeeding practices, by area, and survey year

Breastfeeding status	2002		2004	
	Intervention area	Non-intervention area	Intervention area	Non-intervention area
Not breastfed	0.6	0.3	0.3	1.2
Put on breast within 1 hour of birth	38.6	32.7	76.2	46.7
Put on breast in 1–24 hour(s)	45.3	32.4	20.1	22.6
Put on breast after 1 day	15.2	34.5	3.4	29.1
Do not remember	0.3	-	-	0.3
Colostrum given	86.3	91.7	96.5	93.5
Nothing given before giving breastmilk	36.8	40.5	81.2	36.8
Total number	2,989	336	2,787	323

### Knowledge of newborn danger signs

Most (94.6%) mothers were aware of at least two neonatal danger signs that require immediate medical care (Table 4). Knowledge of two or more neonatal danger signs increased by 3.4%, i.e. from 91.2% in 2002 to 94.6% in 2004. Mothers’ knowledge of three or more neonatal danger signs increased from 39% in 2002 to 55.4% in 2004. Table 5 shows the percentage of mothers by their knowledge about neonatal danger signs that require immediate medical care. The major danger signs of the newborn within seven days of delivery, as perceived by the respondents, were: fever (81.3%), difficult or fast breathing (75.2%), yellow colouration of the palm and sole–jaundice (36.6%), poor sucking or feeding (26.6%), redness and discharge around the umbilicus/cord stump (25.2%), and convulsion (24.3%).

**Table 4.** Knowledge of newborn danger signs/symptoms*

Knowledge of danger signs of infant**	2002		2004	
	Intervention area	Non-intervention area	Intervention area	Non-intervention area
At least two (2+) signs	91.4	44.4	94.6	81.5
At least three (3+) signs	39.0	34.5	55.4	21.4
Total number	2,989	336	2,787	323

*Poor sucking or feeding/unable to suck, frequent watery stools or stools with blood or mucus, difficult or fast breathing/cold, fever, redness and discharge around the cord, red-swollen eyes with discharge, yellow colour on the palm and sole (jaundice), baby is cold (hypothermia), skin colour of the palm/soles–blue, skin lesions (or blisters), and convulsions

**Unprompted response

**Table 5.** Percentage of mothers by their knowledge about postnatal danger signs that require immediate medical attention*

Knowledge of symptoms**	2002		2004	
	Intervention area	Non-intervention area	Intervention area	Non-intervention area
At least two (2+) signs	47.1	26.8	64.3	23.5
At least three (3+) signs	2.7	0.6	12.3	0.7
Total number	2,989	336	2,787	323

*Fever, excessive bleeding, discharge with bad smell, shivering, severe pain in lower abdomen, severe headache/blurred vision, retained placenta, problem in breast, prolapsed uterus, and ruptured vagina

**Unprompted response

### Knowledge of postnatal maternal danger signs

Table 5 summarizes that knowledge on postnatal maternal danger signs requiring immediate medical attention remained low in the non-intervention area. In the intervention area, knowledge on at least two danger signs increased from 47.1% in 2002 to 64.3% in 2004. According to the endline survey data, the major postnatal danger signs that the mothers were aware of included: excessive bleeding (68.8%), severe pain in lower abdomen (41.0%), fever (40.9%), and severe headache/blurred vision (30.7%). A significant proportion of the mothers perceived that convulsion (55.1%) and weakness/faintness (41.2%) were also postnatal danger signs.

About 64% of the mothers were aware of at least two danger signs in the postpartum period that require immediate medical attention. About 12.3% were aware of at least three danger signs. In estimating knowledge of two, three, or more postpartum danger signs, or symptoms, such as fever, excessive bleeding, discharge with bad smell, shivering, severe pain in lower abdomen, severe headache/blurred vision, retained placenta, prolapsed uterus, problem in breast, and ruptured vagina, were considered.

**Fig. 2.**
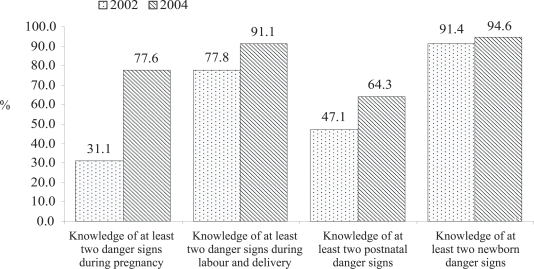
Comparison of mothers' knowledge of at least two maternal danger signs during pregnancy, labour and delivery, and postnatal period and of the newborn

Knowledge of the mothers about at least two postpartum danger signs increased by 17%, i.e. from 47.1% in 2002 to 64% in 2004. Figure 2 shows the comparison of knowledge of at least two danger signs during pregnancy, labour and delivery, postnatal, and newborn periods.

### Postnatal maternal and newborn care

**Fig. 3.**
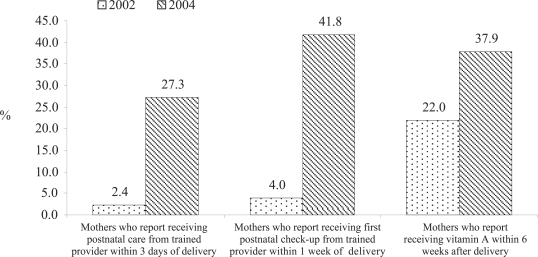
Postnatal maternal check-up from clinically-trained providers* and vitamin A supplements

Table 6 presents data on physical check-up/advice for mothers after delivery and about care providers from the SNL project areas. About 62% of the mothers received healthcare and/or advice at any time after delivery from care providers. Of them, 54.8% received care from skilled providers, such as doctor, nurse/midwife, Family Welfare Visitor, NGO paramedic, Medical Assistant, and Sub-Assistant Community Medical Officer. Postnatal check-up/advice increased significantly (p=0.01) since the baseline survey. It increased by 37.5%, i.e. from 24.2% in 2002 to 61.7% in 2004. Postnatal check-up by clinically-trained providers within three days of delivery showed a significant (p=0.01) rise at 27.3% in 2004 from 2.4% in 2002 and check-up within one week of delivery increased from 4.0% in 2002 to 41.8% in 2004 (Fig. 3).

**Table 6.** Percentage of mothers receiving postnatal care (check-up/advice) by service provider

Survey year/SNL project area		Received health service/advice	No. of mothers	Heath service/advice provided by				
				Total skilled	Total trained	Village doctor	Others	No. of mothers
2002	Intervention area	24.2	2,989	60.7	61.5	33.3	6.4	799
	Non-intervention area	7.1	336	83.3	83.3	16.7	0.0	24
2004	Intervention area	61.7	2,787	54.8	87.5	12.3	2.1	1,742
	Non-intervention area	11.4	323	64.7	70.6	26.5	2.9	37

*Doctor, nurse/midwife, Family Welfare Visitor, non-governmental paramedic, Medical Assistant, Sub-Assistant Community Medical Officer, Family Welfare Assistant/Health Assistant, and care providers of equivalent cadres

**Fig. 4.**
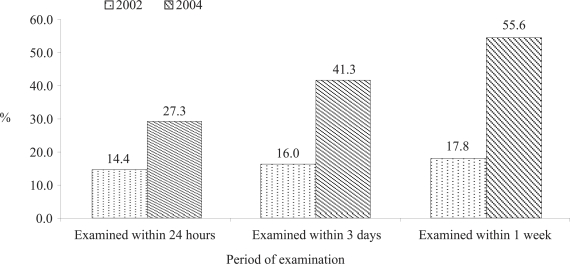
Examination of newborns

Figure 4 shows that newborn check-up immediately after birth (within 24 hours of birth) increased by 12.9% percentage points, i.e. from 14.4% in 2002 to 27.3% in 2004. This also suggests a significant (p=0.01) improvement in newborn-care practice in the SNL project areas.

## DISCUSSION

Despite the significant improvement in the child health and safe motherhood interventions, maternal mortality (320 per 1,000 livebirths), perinatal mortality (65 per 1,000 pregnancies), and neonatal mortality (41 per 1,000 livebirths) still remain a challenge in achieving the Millennium Development Goals in Bangladesh (2). Most newborn deaths are preventable through affordable interventions (6). To address the high burden of maternal and newborn deaths in Bangladesh, focused care must be available during pregnancy, labour/delivery and postpartum periods.

Culturally-appropriate behaviour change communication strategies can increase rates of immediate and exclusive breastfeeding (7). Although breastfeeding practice in Bangladesh is almost universal, putting the newborn to the breast within one hour of birth and within 24 hours of birth were estimated at 38.6% and 45.3% respectively in the 2002 baseline survey. The targeted messages specifically on timing of initiation of breastfeeding through different common channels played a catalytic role in improving the practices. Breastfeeding within an hour increased to 76.2% (p=0.01). Exclusive breastfeeding practices increased from 44.5% in 2002 to 71.0% in 2004 among children aged less than six months. Of babies aged less than one month, 87.6% were exclusively breastfed. These estimates are higher than the national estimates. The BDHS 2004 estimated exclusive breastfeeding among infants aged less than two months at 55% and among children aged less than six months at 36.4% (2).

Women may experience a number of problems associated with childbirth in the postpartum period, i.e. six weeks following delivery. Since the movement of mother and baby is culturally restricted for about 40 days after delivery (8), complications can be detected and treated through proper follow-up visits by health workers to women in the postpartum period. Less than one in five newborns is checked by a health professional within six weeks of delivery. Nationally, only 12% of babies receive a postnatal check-up by a trained health provider within the first two days of delivery (2). Eighteen of the mothers received a postnatal check-up from a trained health service provider within 42 days of delivery, and most (14.5%) check-ups were received within the first two days (2). Of mothers who did not deliver at a facility, only 8% received postnatal care from medically-trained provider (2). The most notable success of the SNL initiative was in postnatal care. In the SNL intervention areas, the strategy was to have a trained healthcare provider visit the postpartum mother at home. Check-up and counselling at any time during the postnatal period were estimated at 61.7%, which is 37.5% higher than in the baseline estimate (24.2%). Home-visits by trained health workers during the postnatal period using job-aid helped track mothers and increased coverage. Since the BDHS 1999–2000, the proportion of mothers receiving a postnatal check-up from a medically-trained provider has increased from 14% in 2002 to 18% in 2004 (2).

Postnatal check-up by clinically-trained providers within three days of delivery showed a significant rise at 27.3% (p=0.01) in 2004 from 2.4% in 2002. Counselling during postnatal check-up on at least two of postnatal complications and reproductive health issues was estimated at 92% which is 19.3% higher than in 2002 (73%) (p=0.01).

It is also encouraging to note that knowledge of the mothers on at least two postnatal danger signs increased by 17.2%, i.e. from 47.1% in 2002 to 64.3% in 2004. Counselling during consultation on at least two of keeping baby warm, breastfeeding, and newborn danger signs was estimated at 93.1% which is 7.5% higher than (p=0.01) the 2002 estimate. Awareness of potentially life-threatening conditions during pregnancy, during delivery, or after delivery is low among Bangladeshi women (3). Mothers’ knowledge of three or more newborn danger signs was recorded at 55.4% in the SNL intervention areas which also showed 16.4% increase since 2002.

Most neonatal deaths usually occur in the first 24 hours of life, and three-quarters of neonatal deaths occur in the first week after birth (1). Postnatal check-up within six hours of delivery (as recommended by the World Health Organization) may not be realistic in many communities but postnatal check-up within 72 hours should be made compulsory to reduce deaths of mothers and neonates.

It may be summarized that the results of the SNL community-based interventions showed remarkable positive changes in practices in most areas of maternal and newborn care. In only a two-year period from 2002 to 2004, significant improvements were achieved in most targeted areas. However, further efforts are needed to increase delivery assistance by trained and skilled providers. Continued efforts are also needed to strengthen the follow-up system so that the progress that has already been achieved in different areas of maternal and newborn-care practices is sustained and improved further.

The coverage of postnatal maternal and newborn care by skilled or trained service providers at proper time needs to be increased substantially. Greater emphasis should be given to these areas with properly-designed awareness-building activities and by creating opportunities for quality care. Community networks, such as Union Parishads, village health committees, and community support groups, also have an important role to play to ensure postnatal care services and appropriate practices. Training, orientations, and support systems—in need of national-level momentum—require substantial strategic investment in maternal and child health programmes. Door-to-door visits by the community health workers, using community registers as job-aids, were an effective approach to identifying pregnant women and following them through pregnancy to the postnatal period. Training community health workers on essential newborn and maternal care improved early postnatal care at the household level and raised awareness on appropriate maternal and newborn-care practices.

The current infrastructure and programme strategies of the Ministry of Health and Family Welfare of the Government of Bangladesh are conducive to address immediate and early postnatal care. If early postnatal care for mothers and newborns is incorporated into the national-level programmes, such as community-based skilled birth attendants, emergency obstetric care (both basic and comprehensive), community-based Integrated Management of Childhood Illness, maternal and child health, National Nutrition Programme (NNP), more mothers and newborns are likely to be benefited. Widespread national awareness-building campaigns on postnatal maternal and neonatal danger signs and healthy community-based practices are also needed. The Government, NGOs, professional organizations, and communities need to act together to save newborn babies and their mothers.

The SNL programme demonstrated that incorporation of essential newborn-care components into the existing infrastructure would improve newborn and maternal care in a short of time. Although the effect of this improvement did not correlate with neonatal mortality due to the brief duration of implementation, we now know from different global research and interventions that reducing neonatal mortality requires improving community and household-level practices of maternal and newborn care simultaneously.

Taken together, the data in this study suggest that project interventions are associated with health-outcome improvements. The success of ensuring immediate and early postnatal care of mothers and newborns within three different health-delivery systems of BRAC, CARE, and BPHC shows that the existing service-delivery and communication strategies can be adapted to include essential newborn-care components. For wider coverage, programmes implementing at scale need to incorporate essential newborn-care components and test the effectiveness. This needs to be speeded up if we aim to achieve the Millennium Development Goals by 2015.

Further research is necessary to determine the effectiveness of individual postnatal care components, such as neonatal resuscitation, immediate care of new borns, skin-to-skin contact, early initiation of breastfeeding, special care of low-birth-weight babies, and management and referral of danger signs. Apart from ensuring the state-of-the-art tool, this will also provide guidelines as to which components are more suitable for rapid scaling up and have the most potential for impact at scale.

## ACKNOWLEDGEMENTS

Saving Newborn Lives initiative of Save the Children-USA is supported by the Bill and Melinda Gates Foundation. Associates for Community and Population Research produced the survey data with the field support from the SNL partners—BRAC, CARE, and BPHC. The authors also acknowledge the technical help of the representatives from SNL, Washington DC Office, Directorate General of Health Services, Directorate General of Family Planning, National Institute of Population Research and Training, ICDDR,B, Bangladesh Perinatal Society, Diabetic Association of Bangladesh, and United States Agency for International Development which were actively involved in the evaluation process.
